# Awareness of Hospice Care Among Rural Residents: Findings from Social Determinants of Health Framework

**DOI:** 10.1093/geroni/igab046.2849

**Published:** 2021-12-17

**Authors:** Yan Luo, Hyunjin Noh, Lewis Lee, Hee Lee

**Affiliations:** 1 The University of Alabama, Tuscaloosa, Alabama, United States; 2 University of Alabama, Tuscaloosa, Alabama, United States; 3 Univeristy of Alabama, Tuscaloosa, Alabama, United States; 4 Univeristy of Alabama, Tuacaloosa, Alabama, United States

## Abstract

Although the demand for hospice care increases as our society ages, the awareness of hospice care among adults in the southern rural region of the US has not been documented. This study aims to assess the rate of hospice care awareness among rural residents living in the Black Belt Region and examine social determinants of health (SDH) associated with the awareness. A cross-sectional survey was conducted among a convenient sample living in rural Alabama (N=182, age=18-91). Participants’ awareness of hospice care, demographic characteristics (i.e., age, gender), and SDH (i.e., financial resources strain, food insecurity, education and health literacy, social isolation, and interpersonal safety) were assessed. Lastly, a binary logistic regression was used to examine the association between SDH and awareness of hospice care among participants while controlling for demographic characteristics. The majority of participants had heard of hospice care (82.4%), and older participants (over 50 years old) were more likely to report having heard of hospice care (OR=7.35, p<0.05). Participants reporting worries about stable housing (OR=0.05, p<0.05) and higher social isolation were less likely to have heard of hospice care (OR=0.53, p<0.05), while participants with higher health literacy had a higher likelihood to have heard of it (OR=2.60, p<0.01). Our study is the first study assessing the status of hospice care awareness among residents living in the Black Belt Region. This study highlighted that factors including age and certain SDH (i.e., housing status, health literacy, and social isolation) might be considered in the intervention to improve hospice care awareness.

